# Generalizing the central dogma as a cross-hierarchical principle of biology

**DOI:** 10.1098/rstb.2024.0296

**Published:** 2025-10-02

**Authors:** Nobuto Takeuchi, Kunihiko Kaneko

**Affiliations:** ^1^ School of Biological Sciences, The University of Auckland, Auckland, New Zealand; ^2^ Universal Biology Institute, The University of Tokyo, Tokyo, Japan; ^3^ Niel Bohr Institute, The University of Copenhagen, Copenhagen, Denmark

**Keywords:** origins of life, reproductive division of labour, spontaneous symmetry breaking, multilevel selection, Price equation, prebiotic evolution

## Abstract

The central dogma of molecular biology, as originally proposed by Crick, asserts that information passed into protein cannot flow back out. This principle has been interpreted as underpinning modern understandings of heredity and evolution, implying the unidirectionality of information flow from nucleic acids to proteins. Here, we propose a generalization of the central dogma as a division of labour between the transmission and expression of information: the transmitter (nucleic acids) perpetuates information across generations, whereas the expressor (protein) enacts this information to facilitate the transmitter’s function without itself perpetuating information. We argue that this generalization offers two benefits. First, it provides a unifying perspective for comparing the central dogma to analogous divisions of labour observed at vastly different biological scales, including multicellular organisms, eukaryotic cells, organelles and bacteria. Second, it offers a theoretical framework to explain the central dogma as an outcome of evolution. Specifically, we review a mathematical model suggesting that the central dogma originates through spontaneous symmetry breaking driven by evolutionary conflicts between different levels of selection. By reframing the central dogma as an informational relationship between components of a system, this generalization underscores its broader relevance across the biological hierarchy and sheds light on its evolutionary origin.

This article is part of the theme issue ‘Origins of life: the possible and the actual’.

## 1. Introduction

The central dogma of molecular biology is one of the most widely known yet frequently misunderstood concepts in biology. Many think it means information flows from DNA to RNA to protein—since this is how it is taught in textbooks [1–4]. However, Crick’s original definition is, ‘once “information” is passed into protein it cannot get out again’ ([5], 138–163). This means, ‘the transfer of information from nucleic acid to nucleic acid, or from nucleic acid to protein may be possible, but transfer from protein to protein, or from protein to nucleic acid is impossible’, where information means ‘the precise determination of sequence, either of bases in the nucleic acid or of amino acid residues in the protein’ ([5], 138–163). Thus, while the central dogma rules out the reading of information from protein, it does not preclude reverse transcription, a process that is often misconceived as reversing the central dogma [6,7].

The central dogma has been interpreted as underpinning the modern understanding of biological evolution, in particular, by providing the molecular basis for the non-inheritance of acquired characters. For example, Monod highlighted the central dogma’s role in establishing the independence of the genetic message from external influences, stating [8]:

Since the whole Darwinian concept is based on change through the inheritance of new traits, on selection pressing on a somewhat varied population, so long as you could not say exactly how inheritance occurred, physically, and what the generator of variety was, chemically, Darwinism was still up in the air. So, what molecular biology has done, you see, is to prove beyond any doubt but in a totally new way the complete independence of the genetic information from events occurring outside or even inside the cell—to prove by the very structure of the genetic code and the way it is transcribed that no information from outside, of any kind, can ever penetrate the inheritable genetic message. This was believed but never proved until the structure of DNA and the mechanisms of protein synthesis were understood. This was what Francis called the Central Dogma: no information goes from protein to DNA.

Maynard Smith also wrote ([9], 177–194):

I think that the non-inheritance of acquired characters is a contingent fact, usually but not always true, not a logical necessity. Insofar as it is true, it follows from the ‘central dogma’ of molecular biology, which asserts that information travels from nucleic acids to proteins, but not from proteins to nucleic acids.

These quotes display a subtle shift in the conceptualization of the central dogma in the context of evolution. Crick defined the central dogma as the impossibility of retrieving information from protein, focusing on a property of a single type of molecule. By contrast, Monod and Maynard Smith reconceptualized the central dogma as the irreversibility of information flow from nucleic acids to proteins, focusing on a relationship between different types of molecules. This shift of focus from the property of a single molecule to the relationship between multiple molecules can be furthered to formulate a beneficial abstraction of the central dogma, as discussed below.

## 2. The central dogma as a division of labour between information transmission and expression

We propose to abstract the central dogma as a division of labour between the transmission and expression of information. The transmitter perpetuates information by maintaining persistent lines of descent, whereas the expressor enacts this information to facilitate the transmitter’s function without directly perpetuating it. This division of labour abstracts the central dogma, with nucleic acids as the transmitter and protein as the expressor. This division implies that the flow of information from nucleic acids to protein is irreversible—if it were reversible, protein would extend its lines of descent interwoven with those of nucleic acids, perpetuating information. However, this division does not exclude the reading of information from protein, generalizing the scope of the central dogma. For example, the replication of protein sequences (i.e. protein-to-protein information transfer) is permissible as long as it remains transient and does not produce persistent lines of descent.^1^ In addition to generalizing the central dogma, this division also introduces an additional attribute that is not mentioned in the central dogma: protein enacts information in nucleic acids to help them perpetuate this information.

The division of labour between information transmission and expression represents a specialized form of the reproductive division of labour. The reproductive division of labour means that some individuals within a social group specialize in reproduction, whereas others perform cooperative tasks such as foraging or defence [10]. However, individuals performing cooperative tasks may sometimes become reproducers, for example, when pre-existing reproducers die [11]. By contrast, the transmitter–expressor division of labour imposes a constraint that the expressor does not become the transmitter, ensuring the unidirectionality of information transfer from the transmitter to the expressor.

Abstracting the central dogma as the division of labour between information transmission and expression offers two benefits. First, it provides a unifying perspective that connects the central dogma to analogous divisions of labour observed across vastly different biological scales. Second, it offers a theoretical framework to explain the central dogma as a logical consequence of evolution, rather than a contingent historical fact, as suggested by Maynard Smith. These advantages are explored in the following sections.

## 3. The central dogma as a particular instance of the cross-hierarchical principle

The central dogma is one example of the division of labour between information transmission and expression. This division has evolved at nearly every conceivable level of biological individuality. Below, we explore how this division manifests across biological hierarchies, as summarized in table 1 (which provides a concise overview, allowing readers to proceed directly to the next section if they wish).

**Table 1.** The division of labour between the transmission and expression of information occurs at distinct biological scales, illustrating the universality of this pattern across the biological hierarchy. (The central dogma of molecular biology is an example of this division at the molecular level (bottom row). *S. coelicolor*, *Streptomyces coelicolor*.*)*

biological scale (elements in a group)	information transmitter	information expressor	division of labour between information transmission and expression
multicellular organisms in a eusocial colony	queens	workers	queens lay eggs. Workers perform tasks, such as foraging and brood care to enhance queens’ reproductive success. Workers are sterile
eukaryotic cells in a multicellular organism	germline cells	somatic cells	germline cells produce gametes. Somatic cells specialize in functions supporting organismal reproduction. Somatic cell lineages do not produce gametes
organelles (nuclei) in a ciliate cell	micronuclei	macronuclei	micronuclei are capable of meiosis. Macronuclei are transcriptionally active. Macronuclei derive from micronuclei via genome fragmentations and truncations
bacterial cells in a colony of *S. coelicolor*	wild-type cells	mutant cells	the wild-type replicates substantially faster than the mutants. Mutants produce antibiotics. Mutants are produced via large-scale genome truncations
molecules in a cell	nucleic acids	protein	the central dogma of molecular biology. Nucleic acids transmit information across generations. Proteins provide catalysis. No reverse translation occurs

At the level of multicellular organisms, the division of labour between information transmission and expression is exemplified by the differentiation between queens and workers in eusocial animals, such as bees and ants [10,12,13]. Queens lay eggs that develop into workers or next-generation queens, perpetuating genetic information. By contrast, workers are sterile and focus on tasks supporting the queens’ reproduction, such as foraging for food, defending the nest and caring for the brood. Therefore, queens are the transmitter, while workers are the expressor.

Another example of this division of labour at the level of multicellular organisms is provided by siphonophores, a class of colonial marine invertebrates related to corals and jellyfish [13,14]. These animals display a differentiation between reproductive and non-reproductive ‘zooids’, which are the clonal units that compose a colony and are homologous to the individuals of solitary jellyfish. In many siphonophores (specifically in the Codonophora group), a batch of zooids clonally develops from each bud that is sequentially generated in the colony’s growth zone [15]. Each batch of zooids then differentiates into a defined organization of reproductive and non-reproductive zooids. Reproductive zooids produce gametes, while non-reproductive zooids are specialized for feeding, defence, etc. Therefore, the growth zone, along with its descendant reproductive zooids, is the transmitter, while the non-reproductive zooids are the expressor. However, to our knowledge, it is unknown whether the differentiation between reproductive and non-reproductive zooids is irreversible.

At the eukaryotic cell level, the division of labour between information transmission and expression is exemplified by the differentiation between germline and somatic cells in multicellular organisms [13,16,17]. Germline cells produce gametes, perpetuating genetic information, whereas somatic cells perform functions that support the germline’s dissemination of gametes. While some somatic cells, such as somatic stem cells, are capable of replication (i.e. soma-to-soma information transmission), they do not differentiate into germline cells and thus do not produce gametes. This irreversible differentiation ensures the unidirectionality of information transfer. Therefore, germline cells are the transmitter, while somatic cells are the expressor.

We add that germline–soma distinction should be distinguished from early germline segregation, which is a subset of germline–soma distinction [18,19]. Early germline segregation refers to the separation of germline cell lineages from somatic cell lineages at early stages of development, thereby preventing germline cells from contributing to the production of somatic cells during most of the organism’s lifetime. By contrast, germline–soma distinction does not depend on when—or even whether—germline cell lineages are segregated from somatic cell lineages. For example, in sponges, which are a basal group of metazoans, the stem-cell system consisting of archaeocytes and choanocytes is capable of producing all cell types, including somatic cells and gametes, throughout sponges’ lifetime [20,21,]. By contrast, somatic cells cannot differentiate into these stem cells or produce gametes. Therefore, sponges display germline–soma distinction but lack germline segregation [19]. In fact, early germline segregation occurs only in a few taxa, such as vertebrates and insects, whereas germline–soma distinction is widespread across metazoans [18,19].

At the organellar level, the transmitter–expressor division of labour is exemplified by the differentiation between micronuclei and macronuclei in ciliates, a group of unicellular predators including *Tetrahymena* and *Paramecium* [22–24]. The micronucleus contains two copies of each chromosome (diploid). By contrast, the macronucleus contains numerous copies of subchromosomal DNA molecules, which are derived from the chromosomes of the micronucleus through genome-wide site-specific recombination, fragmentation and truncation—processes that are likely to be irreversible [22]. The micronucleus is transcriptionally silent, whereas the macronucleus is active [22]. High DNA copy numbers in the macronucleus are considered to enhance transcriptional throughput, supporting large cell sizes, which are likely to be advantageous for predatory lifestyles [24]. Ciliates reproduce both sexually and asexually. During asexual reproduction, the micronucleus replicates through mitosis, while the macronucleus replicates through ‘amitosis’, a process that stochastically distributes DNA molecules to daughter cells after DNA replication [22]. During sexual reproduction, the micronucleus replicates through meiosis, and one of the resulting micronuclei is exchanged with another cell via conjugation [23]. Micronuclei from the two cells then fuse, forming the micronucleus of the next generation. Meanwhile, the macronucleus is completely destroyed and de novo reconstituted through the replication and genome-wide restructuring of the new micronucleus [23]. This process ensures that the macronucleus is unable to transmit genetic information through sexual reproduction. Therefore, the micronucleus is the transmitter, while the macronucleus is the expressor.

At the bacterial cell level, the transmitter–expressor labour division is exemplified by the differentiation between the wild-type and mutant subpopulations in a colony of *Streptomyces coelicolor* [25]. The mutant subpopulations are derived from the wild-type through large-scale genome truncation, which deletes approximately $10^{3}$ genes—a process that is likely to be irreversible. The mutant subpopulations display enhanced antibiotic production but reduced spore production compared to the wild-type. Therefore, the wild-type is the transmitter, while the genome-truncated mutants are the expressor. Another example at this level is the ‘germ–soma’ distinction in bacterial symbionts of multiple insect species [26–28].

The examples outlined above illustrate that the division of labour between information transmission and expression is an organizational principle observed across many distinct biological scales, with the central dogma as an example at the molecular level. Many of these examples are associated with a ‘fraternal’ major evolutionary transition, a process in which individuals stay together after reproduction to form a higher level unit of reproduction [13,29–31]. For example, germline–soma division occurs in multicellular organisms, where cells remain together after replication, and queen–worker division occurs in eusocial colonies, where individuals stay in their natal nests after maturation. The central dogma can be conceived as an evolutionary analogue of these divisions, corresponding to the major transition in which replicating molecules are grouped into collectives, such as protocells [32]. This perspective raises the question of whether the origin of the central dogma can be explained by a general evolutionary mechanism related to major transitions, a question we discuss in §4.

## 4. The central dogma as a consequence of multilevel selection

Why does the central dogma hold? Crick states that it is highly improbable, for stereochemical reasons, that protein-to-protein information transfer can occur with the same simplicity as DNA-to-DNA transfer [7]. Furthermore, Crick notes that protein-to-RNA (or DNA) information transfer would require complicated machinery that is entirely distinct from the one used for RNA-to-protein information transfer and that there is no reason to believe that such machinery might be needed by the cell [7].

Koonin suggests that protein folding is the fundamental cause of the central dogma [33]. Koonin argues that protein folding prevents the accurate retrieval of sequence information because amino acid residues that are distant in the primary structure can become proximal in the tertiary structure. Thus, retrieving information from protein requires denaturation. However, denaturation generates misfolded globules, which are toxic for the cell and thus need to be quickly degraded. Consequently, protein folding irreversibly blocks the reading of amino-acid sequence information, causing the central dogma [33].

The hypotheses of Crick and Koonin described above are rooted in the conceptualization of the central dogma as a chemical property of single molecules: once information is passed into a protein, it cannot get out again. Reconceptualizing the central dogma as a division of labour between the transmission and expression of information offers an alternative framework to explain its origin [32]. This framework describes how evolution drives the differentiation of molecules into information transmitters (‘genes’) and expressors (‘enzymes’), starting with undifferentiated molecules serving both functions. This framework aligns with the RNA world hypothesis, which posits that RNA fulfilled the dual roles of genes and enzymes before DNA and protein evolved. However, while the RNA world hypothesis emphasizes the unique chemistry of RNA, this framework focuses on informational relationships between molecules and is thus agnostic to their specific chemical properties.

### (a) Mathematical model

To explore the evolution of gene–enzyme differentiation, we review the mathematical model introduced by Takeuchi & Kaneko [32]. The model assumes molecules that are capable of functioning as both templates for replication and catalysts for template replication, as is also assumed in the RNA world hypothesis. Initially, all molecules are assumed to function as templates and catalysts equally well. The model explores whether and how evolution leads to gene–enzyme differentiation, which is defined by the emergence of the following two asymmetries:

— *catalytic asymmetry*: enzymatic molecules provide catalysis, whereas genic molecules do not; and
— *cransmission asymmetry*: genic molecules transmit information across generations, whereas enzymatic molecules do not.

To investigate this question, the model incorporates the following assumptions, the rationale for which will be explained later:

(i) providing catalysis is an ‘altruistic’ trait;
(ii) the population of molecules is ‘stage-structured’; and
(iii) ‘multilevel selection’ operates.

The first assumption, that providing catalysis is altruistic, is due to the following trade-off: providing catalysis requires a molecule to fold into specific secondary and tertiary structures, whereas serving as a template requires these structures to unfold [34,35]. Therefore, providing catalysis promotes the replication of other molecules at the expense of the replication of self, thus representing an altruistic trait [36–39].

The second assumption, that the population is stage-structured, posits that molecules carrying equivalent information can exist in different stages (or states) and express this information differently depending on the stage. For example, within a population of molecules carrying equivalent information, those in one stage might provide catalysis, while those in the other stage might not. This stage-dependent catalysis abstracts the relationship between genes and enzymes: they carry equivalent information related through the genetic code, but only enzymes provide catalysis. Thus, stage-dependent catalysis allows the evolution of catalytic asymmetry, one of the two asymmetries that characterize gene–enzyme distinction (the other, transmission asymmetry, will be described later).

**Figure 1.**
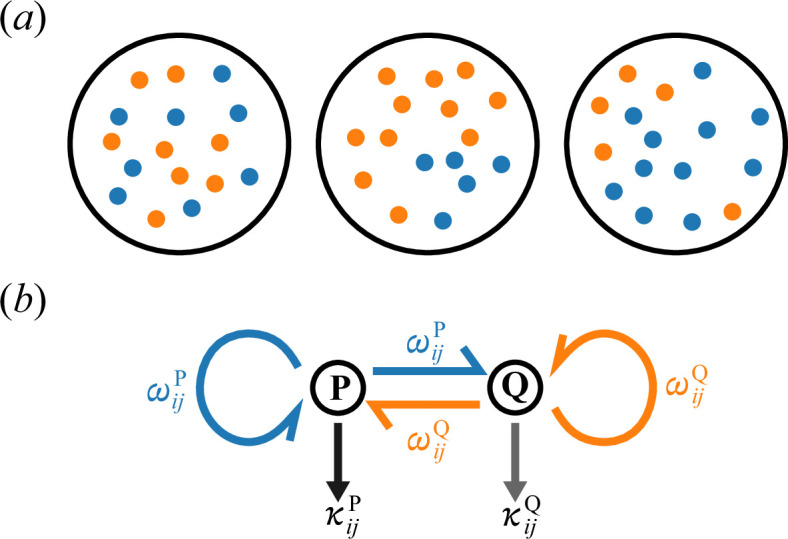
The model of gene–enzyme differentiation. (*a*) Replicating molecules (dots) are compartmentalized into protocells (circles), allowing multilevel selection to operate. (*b*) Molecule j in stage c∈{P,Q} in protocell i has catalytic activity $\kappa_{ij}^{c}$ (vertical arrows) and acts as a template to be replicated (circular arrows) or transcribed (horizontal arrows) at an identical rate $\omega_{ij}^{c}$. This rate increases with an increase in the average catalytic activity molecules in protocell i (denoted as $\left\langle \kappa_{i\tilde{j}}^{c}\right\rangle$ in the main text), but decreases with an increase in the molecule’s own catalytic activity $\kappa_{ij}^{c}$, reflecting the trade-off between catalysing and templating functions.

The third assumption, that multilevel selection operates, posits that the population of molecules is compartmentalized into primordial cells, or protocells for short (figure 1*a*), and that natural selection operates at two levels:

(i) within-cell selection, which arises from variations in replication rates across different molecules within the same cell; and
(ii) between-cell selection, which arises from variations in the average replication rates of molecules across different cells.

Multilevel selection is necessary because the provision of catalysis, which is altruistic, cannot evolve in a well-mixed population, where each molecule interacts equally likely with all others [40].

A simple mathematical model incorporating the above assumptions can be formulated as follows [32]:

(i) molecules exist in one of two stages, denoted as P and Q (figure 1*b*);
(ii) a molecule j within protocell i has a catalytic activity $\kappa_{ij}^{c}$ when in stage c (figure 1*b*);
(iii) the rate at which the molecule acts as a template to produce a new molecule decreases with an increase in its catalytic activity $\kappa_{ij}^{c}$, reflecting the trade-off between catalysing and templating functions;
(iv) this production rate increases with an increase in the average catalytic activity of molecules in protocell i, denoted as $\left\langle \kappa_{i\tilde{j}}^{\text{P}}\right\rangle$ and $\left\langle \kappa_{i\tilde{j}}^{\text{Q}}\right\rangle$, where the tildes in the angular brackets mark the indices over which the average is taken (this notational convention also applies to other symbols);
(v) a newly produced molecule either remains in the same stage as the template molecule (considered ‘replication’) or transition to the other stage (considered ‘transcription’). For simplicity, replication and transcription occur at the same rate, denoted $\omega_{ij}^{c}$ (figure 1*b*). No other types of stage transition occur; and
(vi) for simplicity, the mutation of molecules and the growth and division of protocells are not explicitly modelled. Instead, it is assumed that variances in catalytic activity $\kappa_{ij}^{c}$ within protocells (i.e. the variance of $\kappa_{ij}^{c}$ across j within i, averaged over all i) and between protocells (i.e. the variance of $\left\langle \kappa_{i\tilde{j}}^{c}\right\rangle$ across i) are maintained at $\sigma_{\text{w}}^{2}$ and $\sigma_{\text{b}}^{2}$, respectively.

The fitness of a molecule (denoted $\lambda_{ij}$) is defined as the growth rate of a hypothetical population generated through the replication and transcription of this molecule (i.e. its descendants) without mutation. To calculate $\lambda_{ij}$, let $n_{ij}^{c}(\tau )$ be the number of molecules in stage c in this hypothetical population at time τ. Since the number of replication or transcription events per unit time is $w_{ij}^{c}$, the following equation describes the growth of this population:


(4.1) $$\begin{array}{r} \left[\begin{array}{c} n_{ij}^{\text{P}}(\tau +1)\\ n_{ij}^{\text{Q}}(\tau +1) \end{array}\right]=\left[\begin{array}{cc} \omega_{ij}^{\text{P}} & \omega_{ij}^{\text{Q}}\\ \omega_{ij}^{\text{P}} & \omega_{ij}^{\text{Q}} \end{array}\right]\left[\begin{array}{c} n_{ij}^{\text{P}}(\tau )\\ n_{ij}^{\text{Q}}(\tau ) \end{array}\right]. \end{array}$$

The fitness $\lambda_{ij}$ corresponds to the largest eigenvalue of the 2 × 2 matrix on the right-hand side of equation (4.1), which is calculated as:


(4.2) $$\begin{array}{lcr} & \lambda_{ij}=\omega_{ij}^{\text{P}}+\omega_{ij}^{\text{Q}}. & \end{array}$$

### (b) Analysis of the model

While the model incorporates the possibility of catalytic asymmetry as a disparity between $\kappa_{ij}^{\text{P}}$ and $\kappa_{ij}^{\text{Q}}$, it also accounts for transmission asymmetry, represented by a disparity between $\omega_{ij}^{\text{P}}$ and $\omega_{ij}^{\text{Q}}$. To show this, we consider the population of molecules described by equation (4.1). Let $u_{ij}^{c}$ be the number of molecules (including both P and Q) generated through the replication or transcription of molecule j in stage c within protocell i over a time period T. As T→∞, the normalized value of $u_{ij}^{c}$ converges to a quantity known as Fisher’s reproductive value, which quantifies the stage c’s relative contribution to the future population [41]. If a molecule in stage c is responsible for the perpetuation of information—that is, it performs genic function—then $u_{ij}^{c}>0$. If it does not, then $u_{ij}^{c}=0$. Therefore, the evolution of transmission asymmetry corresponds to the evolution of reproductive value asymmetry [32]. Mathematically, these reproductive values are the elements of the left eigenvector associated with the largest eigenvalue $\lambda_{ij}$ of the matrix on the right-hand side of equation (4.1) [41]. Thus, it can be shown that:


(4.3) $$\begin{array}{rl} u_{ij}^{\text{P}} & =\omega_{ij}^{\text{P}},\\ u_{ij}^{\text{Q}} & =\omega_{ij}^{\text{Q}}. \end{array}$$

Equation (4.3) aligns with the intuitive idea that the more frequently a molecule replicates, the greater its contribution to information transmission (however, the specific form of equation (4.3) relies on the simplifying assumption that the rates of transcription and replication are equal).

To summarize, catalytic asymmetry is modelled as a disparity between $\kappa_{ij}^{\text{P}}$ and $\kappa_{ij}^{\text{Q}}$, while transmission asymmetry is modelled as that between $\omega_{ij}^{\text{P}}$ and $\omega_{ij}^{\text{Q}}$. These asymmetries are interdependent because an increase in $\kappa_{ij}^{c}$ reduces $\omega_{ij}^{c}$ owing to the trade-off between catalytic and template functions. In other words, catalytic asymmetry gives rise to transmission asymmetry.

To understand how catalytic activity evolves, we calculate the change in the average catalytic activity of the system per unit time, denoted $\Delta \langle \kappa_{\tilde{i}\tilde{j}}^{c}\rangle$. Since each molecule leaves the number of descendants proportional to its fitness per unit time, the following equation holds:


(4.4) $$\Delta \langle \kappa_{\tilde{i}\tilde{j}}^{c}\rangle =\frac{\sum_{ij}\lambda_{ij}\kappa_{ij}^{c}}{\sum_{ij}\lambda_{ij}}-\langle \kappa_{\tilde{i}\tilde{j}}^{c}\rangle .$$

Under the assumptions that the between-cell variance $\sigma_{\text{b}}^{2}$ and the within-cell variance $\sigma_{\text{w}}^{2}$ of catalytic activity are sufficiently small and that $\kappa_{ij}^{\text{P}}$ and $\kappa_{ij}^{\text{Q}}$ are uncorrelated as i and j are varied, $\Delta \langle \kappa_{\tilde{i}\tilde{j}}^{c}\rangle$ can be approximated as:


(4.5a)Δ⟨κi~j~P⟩≈⟨ωi~j~P⟩(βσb2−γσw2)+⟨ωi~j~Q⟩βσb2⟨λi~j~⟩,(4.5b)Δ⟨κi~j~Q⟩≈⟨ωi~j~P⟩βσb2+⟨ωi~j~Q⟩(βσb2−γσw2)⟨λi~j~⟩.

These approximations hold to the linear terms of the second central moments of $\kappa_{ij}^{c}$, ignoring the nonlinear terms of these moments and the higher order moments (see [32] for details).

Equations (4.5) have the following meaning. Symbols β and γ represent selection pressures at different levels (β≥0 and γ≥0). Specifically, β is the strength of between-cell selection acting on catalytic activity. The positiveness of the terms involving β means that between-cell selection favours an increase in catalytic activity. By contrast, γ is the strength of within-cell selection acting on catalytic activity. The negativeness of the terms involving γ means that within-cell selection favours a decrease in catalytic activity. This negative effect comes from the fact that increasing the catalytic activity of a molecule reduces this molecule’s relative replication rate within a protocell, as providing catalysis is altruistic. Together, these opposing forces cause conflicting multilevel selection, a process in which selection operates antagonistically at two levels.

The terms involving $\left\langle \omega_{\tilde{i}\tilde{j}}^{\text{P}}\right\rangle$ and $\left\langle \omega_{\tilde{i}\tilde{j}}^{\text{Q}}\right\rangle$ in equations (4.5) represent different transmission pathways through which evolution occurs. Specifically, the terms involving $\left\langle \omega_{\tilde{i}\tilde{j}}^{\text{P}}\right\rangle$ represent contributions to evolution through the descendants of P molecules, i.e. through P’s information transmission. By contrast, the terms containing $\left\langle \omega_{\tilde{i}\tilde{j}}^{\text{Q}}\right\rangle$ represent contributions to evolution through the descendants of Q molecules, i.e. through Q’s information transmission.

Equations (4.5) imply a positive feedback loop between the evolution of catalytic asymmetry and that of transmission asymmetry, suggesting spontaneous symmetry breaking. To illustrate this, we begin with a system that is symmetric with respect to both catalysis and transmission: $\left\langle \kappa_{\tilde{i}\tilde{j}}^{\text{P}}\rangle =\langle \kappa_{\tilde{i}\tilde{j}}^{\text{Q}}\right\rangle$ and $\left\langle \omega_{\tilde{i}\tilde{j}}^{\text{P}}\rangle =\langle \omega_{\tilde{i}\tilde{j}}^{\text{Q}}\right\rangle$. In this state, P and Q molecules are functionally identical, so that changes in their catalytic activities are identical too: $\Delta \langle \kappa_{\tilde{i}\tilde{j}}^{\text{P}}\rangle =\Delta \langle \kappa_{\tilde{i}\tilde{j}}^{\text{Q}}\rangle$. Next, we introduce a slight perturbation to catalytic symmetry, owing to mutations or random genetic drift, such that P’s catalytic activity is marginally increased compared with that of Q:


(4.6) $$\begin{array}{lcr} & \langle \kappa_{\tilde{i}\tilde{j}}^{\text{P}}\rangle >\langle \kappa_{\tilde{i}\tilde{j}}^{\text{Q}}\rangle . & \end{array}$$

This increase comes at a cost to P’s templating activity, reducing its transmission compared with Q:


(4.7) $$\begin{array}{lcr} & \langle \omega_{\tilde{i}\tilde{j}}^{\text{P}}\rangle <\langle \omega_{\tilde{i}\tilde{j}}^{\text{Q}}\rangle . & \end{array}$$

The resulting transmission asymmetry further amplifies catalytic asymmetry:


(4.8) $$\begin{array}{lcr} & \Delta \langle \kappa_{\tilde{i}\tilde{j}}^{\text{P}}\rangle >\Delta \langle \kappa_{\tilde{i}\tilde{j}}^{\text{Q}}\rangle , & \end{array}$$

as explained in the next paragraph.

Equation (4.8) follows from the fact that equations (4.5) involve asymmetric coupling between conflicting multilevel selection (i.e. the terms involving β and γ) and different transmission pathways (i.e. the terms involving $\left\langle \omega_{\tilde{i}\tilde{j}}^{\text{P}}\right\rangle$ and $\left\langle \omega_{\tilde{i}\tilde{j}}^{\text{Q}}\right\rangle$). Specifically, in equation (4.5a), γ appears in the term containing $\left\langle \omega_{\tilde{i}\tilde{j}}^{\text{P}}\right\rangle$ but not in the term containing $\left\langle \omega_{\tilde{i}\tilde{j}}^{\text{Q}}\right\rangle$. This means that within-cell selection tends to decrease P’s catalytic activity through P’s descendants but not through Q’s descendants. This is because an increase in the catalytic activity of a P molecule reduces the replication rate of this molecule but not the replication rate of its transcript, Q. Likewise, in equation (4.5b), γ appears in the term containing $\left\langle \omega_{\tilde{i}\tilde{j}}^{\text{Q}}\right\rangle$ but not in the term containing $\left\langle \omega_{\tilde{i}\tilde{j}}^{\text{P}}\right\rangle$, permitting a similar interpretation as above. By contrast, β appears in both terms containing $\left\langle \omega_{\tilde{i}\tilde{j}}^{\text{P}}\right\rangle$ and terms containing $\left\langle \omega_{\tilde{i}\tilde{j}}^{\text{P}}\right\rangle$ in equations (4.5a) and (4.5b). This is because an increase in the average catalytic activity of molecules (whether P or Q) in a protocell increases the replication rate of all molecules in that protocell. This asymmetric coupling between multilevel selection and transmission pathways means that when P is replicated less frequently than Q (i.e. $\langle \omega_{\tilde{i}\tilde{j}}^{\text{P}}\rangle <\left\langle \omega_{\tilde{i}\tilde{j}}^{\text{Q}}\right\rangle$), within-cell selection on P’s catalytic activity through P’s descendants (i.e. $\langle \omega_{\tilde{i}\tilde{j}}^{\text{P}}\rangle \gamma \sigma_{\text{w}}^{2}$) has less impact than within-cell selection on Q’s catalytic activity through Q’s descendants (i.e. $\langle \omega_{\tilde{i}\tilde{j}}^{\text{Q}}\rangle \gamma \sigma_{\text{w}}^{2}$). Therefore, equation (4.8) follows. The result that equation (4.8) follows from equation (4.6) via equation (4.7) indicates a positive feedback loop, whereby catalytic asymmetry and transmission asymmetry reinforce each other.

Whether the feedback loop described in the previous paragraph induces symmetry breaking between P and Q depends on the relative magnitudes of within-cell and between-cell variances, $\sigma_{\text{w}}^{2}$ and $\sigma_{\text{b}}^{2}$, respectively. Specifically, under the assumption that within-cell and between-cell selection pressures are of similar magnitudes (β∼σ), if $\sigma_{\text{w}}^{2}\ll \sigma_{\text{b}}^{2}$, equations (4.5) simplify to:


(4.9a)Δ⟨κi~j~P⟩≈βσb2,(4.9b)Δ⟨κi~j~Q⟩≈βσb2,

up to the linear terms of the second central moments of $\kappa_{ij}^{c}$. These equations indicate that between-cell selection dominates evolutionary dynamics, preventing spontaneous symmetry breaking.

To examine whether spontaneous symmetry breaking occurs when $\sigma_{\text{w}}^{2}$ is sufficiently large relative to $\sigma_{\text{b}}^{2}$, we consider the following specific definition of $\omega_{ij}^{c}$:


(4.10) $$\begin{array}{lrr} & \omega_{ij}^{c}=e^{\left\langle \kappa_{i\tilde{j}}^{\text{P}}\rangle +\langle \kappa_{i\tilde{j}}^{\text{Q}}\right\rangle }\frac{e^{-\kappa_{ij}^{c}}}{\left\langle e^{-\kappa_{i\tilde{j}}^{\text{P}}}\rangle +\langle e^{-\kappa_{i\tilde{j}}^{\text{Q}}}\right\rangle }, & \end{array}$$

where c is P or Q [32]. This definition, which was arbitrarily chosen to balance simplicity and the need to satisfy the model assumptions listed earlier, involves three factors. The first factor $e^{\left\langle \kappa_{i\tilde{j}}^{\text{P}}\rangle +\langle \kappa_{i\tilde{j}}^{\text{Q}}\right\rangle }$ means that $\omega_{ij}^{c}$ increases with an increase in the average catalytic activity in protocell i. The second factor $e^{-\kappa_{ij}^{c}}$ means that $\omega_{ij}^{c}$ decreases with an increase in the molecule’s catalytic activity, representing the cost of providing catalysis. The last factor normalizes this cost so that the per-cell average fitness of molecules $\left\langle \lambda_{i\tilde{j}}\right\rangle$ has a simple expression, namely, $e^{\left\langle \kappa_{i\tilde{j}}^{\text{P}}\rangle +\langle \kappa_{i\tilde{j}}^{\text{Q}}\right\rangle }$.

**Figure 2.**
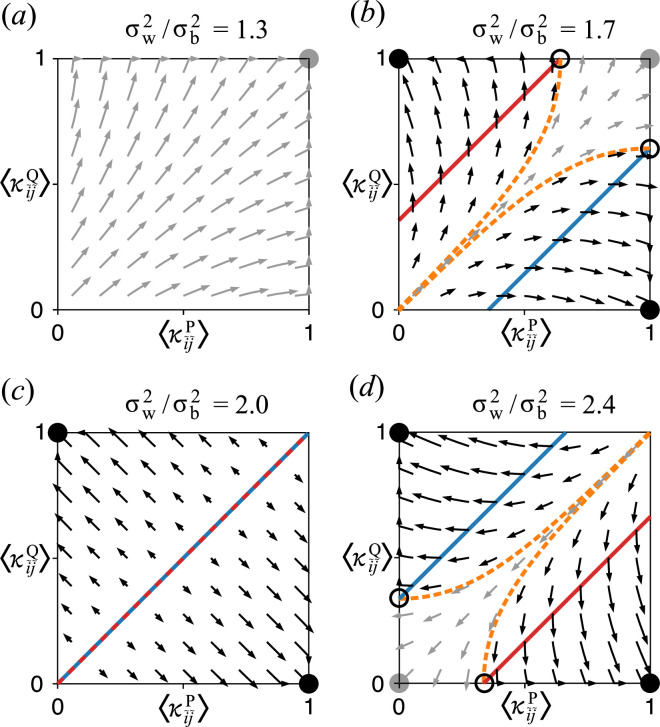
Phase–plane analyses of equations (4.11) for different ratios of $\sigma_{\text{w}}^{2}$ and $\sigma_{\text{b}}^{2}$. To perform these analyses, equations (4.11) were adapted by replacing Δ with time derivative $\frac{\text{d}}{\text{d}\tau }$ and setting $\frac{\text{d}}{\text{d}\tau }\langle \kappa_{\tilde{i}\tilde{j}}^{c}\rangle =0$ when $\langle \kappa_{\tilde{i}\tilde{j}}^{c}\rangle =0$ or 1 so that $\left\langle \kappa_{\tilde{i}\tilde{j}}^{c}\right\rangle$ is bounded. The solid lines represent the nullclines of $\left\langle \kappa_{\tilde{i}\tilde{j}}^{\text{P}}\right\rangle$ (red) and $\left\langle \kappa_{\tilde{i}\tilde{j}}^{\text{Q}}\right\rangle$ (blue). The filled circles indicate symmetric (grey) and asymmetric (black) stable equilibria; the open circles, unstable equilibria. The arrows indicate vector fields leading to symmetric (grey) or asymmetric (black) equilibria. The dashed lines (orange) demarcate the basins of attraction. (*a*) Within-cell variance is so small that between-cell selection completely dominates, maximizing both $\left\langle \kappa_{\tilde{i}\tilde{j}}^{\text{P}}\right\rangle$ and $\left\langle \kappa_{\tilde{i}\tilde{j}}^{\text{Q}}\right\rangle$. (*b*) Within-cell variance is large enough to create stable asymmetric equilibria. However, the equilibrium maximizing both $\left\langle \kappa_{\tilde{i}\tilde{j}}^{\text{P}}\right\rangle$ and $\left\langle \kappa_{\tilde{i}\tilde{j}}^{\text{Q}}\right\rangle$ is still present and stable. Spontaneous symmetry breaking occurs when the initial values of $\left\langle \kappa_{\tilde{i}\tilde{j}}^{\text{P}}\right\rangle$ and $\left\langle \kappa_{\tilde{i}\tilde{j}}^{\text{Q}}\right\rangle$ are sufficiently low. (*c*) The critical point, where the nullclines overlap. (*d*) Within-cell variance is so large that the equilibrium maximizing both $\left\langle \kappa_{\tilde{i}\tilde{j}}^{\text{P}}\right\rangle$ and $\left\langle \kappa_{\tilde{i}\tilde{j}}^{\text{Q}}\right\rangle$ does not exist. Spontaneous symmetry breaking occurs when the initial values of $\left\langle \kappa_{\tilde{i}\tilde{j}}^{\text{P}}\right\rangle$ and $\left\langle \kappa_{\tilde{i}\tilde{j}}^{\text{Q}}\right\rangle$ are sufficiently high. The figure is reproduced from Takeuchi & Kaneko [32].

By the definition in equation (4.10), equations (4.5) can be transformed as:


(4.11a)Δ⟨κi~j~P⟩≈σb2−σw2⟨e−κij~P⟩⟨e−κij~P⟩+⟨e−κij~Q⟩,(4.11b)Δ⟨κi~j~Q⟩≈σb2−σw2⟨e−κij~Q⟩⟨e−κij~P⟩+⟨e−κij~Q⟩,

again up to the linear terms of the second central moments of $\kappa_{ij}^{c}$ [32]. Equations (4.11) were investigated through phase–plane analyses for different ratios of $\sigma_{\text{w}}^{2}$ and $\sigma_{\text{b}}^{2}$, as displayed in figure 2 [32]. These analyses reveal that when $\sigma_{\text{w}}^{2}$ is sufficiently large relative to $\sigma_{\text{b}}^{2}$, P and Q can undergo spontaneous symmetry breaking, differentiating into molecules that provide catalysis but are not replicated or transcribed (‘enzymes’) and those that are replicated and transcribed but do not provide catalysis (‘genes’). This result suggests that the distinction between genes and enzymes can evolve as a consequence of multilevel selection operating on a stage-structured population of replicating catalytic molecules, provided $\sigma_{\text{w}}^{2}$ is sufficiently large relative to $\sigma_{\text{b}}^{2}$.

The condition that $\sigma_{\text{w}}^{2}$ is sufficiently large relative to $\sigma_{\text{b}}^{2}$, by definition, means that the variance of catalytic activity between molecules within protocells is sufficiently large relative to the variance of catalytic activity between protocells. This condition is fulfilled when the number of molecules per protocell is large, the mutation rate of molecules is high or both [32,42]. This condition corresponds to low genetic relatedness between molecules—specifically, this relatedness is equal to $\sigma_{\text{b}}^{2}/(\sigma_{\text{w}}^{2}+\sigma_{\text{b}}^{2})$ [32,42]. Therefore, the model reviewed above predicts that a gene–enzyme distinction evolves when genetic relatedness is low.

The mathematical results described above agree with the computational simulations of an individual-based model that explicitly incorporates chemical reaction, mutation, resource limitation and compartmentalization of molecules [32].

The model reviewed above differs from previous models in two key respects. First, our model explicitly demonstrates the differentiation of molecules into those that transmit information (genes) and those that provide catalysis (enzymes) through spontaneous symmetry breaking. Some of the previous models do not allow any symmetry breaking [37,39]. Other models demonstrate catalytic symmetry breaking but do not demonstrate the unidirectional flow of information between catalysts and non-catalysts [43,44], since they assume that catalytic asymmetry occurs between complementary strands of RNA molecules [45,46]. Another model demonstrates the evolution of this unidirectional information flow but does not demonstrate catalytic symmetry breaking, since it predefines one type of molecules as a catalyst and the other as a non-catalyst [47].

The second key difference with our model concerns a condition for the evolution of gene–enzyme division. Our model predicts that this evolution occurs when relatedness is sufficiently low, whereas the model of Michod [36] predicts that it occurs when relatedness is sufficiently high. This contrast reflects a fundamental difference in the underlying mechanisms for this evolution. Our model relies on the positive feedback loop emerging from conflicting multilevel selection acting on a stage-structured population. By contrast, Michod’s model relies on competition between two species of replicators—catalyst (altruist) and non-catalyst (cheater)—in a subdivided population. Michod’s model predicts that the catalyst is favoured when relatedness is sufficiently high [36]. This outcome is interpreted as indicating that the division of labour between genes and enzymes is favoured when relatedness is sufficiently high [36], although this division is not explicitly included in the model.

Finally, we add that the division of labour between genes and enzymes has been demonstrated to evolve through spontaneous symmetry breaking even when molecules are not compartmentalized into protocells but spatially distributed on a surface [48]. In this case, the division of labour evolves when the diffusion constant of molecules is sufficiently high, a condition that corresponds to low relatedness. This means that the division of labour between genes and enzymes does not necessarily depend on the origin of protocells.

## 5. Evolutionary consequences of the central dogma

The emergence of the division of labour between information transmission and expression can enhance the evolvability of protocells in three ways. First, this division probably induces selection to increase the copy number of enzymes per protocell relative to genes—i.e. numerical asymmetry—because increasing enzyme abundance enhances the productivity of protocells. This trend has been observed in computer simulations [32]. The resulting decrease in the relative copy number of genes amplifies the effects of mutations on the protocell’s phenotype, thereby increasing its evolvability [49]. Specifically, a low gene copy number allows mutations to cause large phenotypic variation, whereas a high gene copy number averages out the effects of mutations owing to the law of large number [49,50]. Notably, similar numerical asymmetry is observed at the other levels of the biological hierarchy, such as small numbers of germline cells per multicellular organism and queens per eusocial colony.

Second, in the context of number symmetry breaking in the generalized central dogma, the replication of minority elements (e.g. DNA) occurs at a significantly slower rate than that of majority elements (e.g. proteins) [49]. More broadly, evolving dynamical systems often exhibit a trend where elements with slower timescales regulate the behaviour of those with faster dynamics. This separation of timescales also facilitates evolution [51,52]. Slower elements typically function as information carriers (genotype), while faster elements act as expressors (phenotype) [53], a principle also explored in the context of multilevel learning [54].

Third, the division of labour between information transmission and expression enhances the evolvability of protocells by enabling the regulation of information expression. Without this division, identical molecules both transmit and express information, forcing information to be expressed whenever it is transmitted. By separating these functions, the division of labour enables control over information expression. This ability probably enhances the cell’s capacity to adapt to changing environments.

## 6. Open questions

Finally, we highlight several open questions that arise from reconceptualizing the central dogma as a division of labour between information transmission and expression.

First, DNA and protein molecules are linked through the genetic code in reality. How does such a symbolic relationship emerge?

Second, the division of labour between information transmission and expression evolved independently across vastly different biological scales. Can this recurrent pattern be explained by the same symmetry-breaking mechanism described above? How does this mechanism differ from those proposed for the evolution of the germline–soma distinction and eusociality [2,55–59]?

Third, the central dogma in biological systems is manifested by DNA and protein, with RNA serving as an intermediary. How do the specific chemical properties and roles of these materials influence or interact with the proposed symmetry-breaking mechanism?

## Data Availability

This article has no additional data.

## Footnotes

^1^
The protein replication discussed in the main text excludes prions. However, prions provide an illuminating thought exercise in relation to our argument. Unlike nucleic acids, prions transmit structural rather than sequence information, yet they can still display the division of labour between information transmission and expression. Suppose a protein has three distinct folds: a natural fold, a template misfold and a catalytic misfold. The template misfold transmits structural information by serving as a ‘template’ to convert the natural fold into additional copies of itself. The catalytic misfold catalyses this structural conversion. The catalytic misfold is generated through the irreversible structural transformation of the template misfold.
